# Machine Learning Prediction of the Redox Activity of Quinones

**DOI:** 10.3390/ma16206687

**Published:** 2023-10-14

**Authors:** Ilia Kichev, Lyuben Borislavov, Alia Tadjer, Radostina Stoyanova

**Affiliations:** 1 Institute of General and Inorganic Chemistry, Bulgarian Academy of Sciences, 1113 Sofia, Bulgaria; ikichev@uni-sofia.bg (I.K.); radstoy@svr.igic.bas.bg (R.S.); 2 Faculty of Chemistry and Pharmacy, University of Sofia, 1164 Sofia, Bulgaria

**Keywords:** quinones, machine learning, ridge regression, decision tree, ensemble methods, density functional theory, organic electrode materials

## Abstract

The redox properties of quinones underlie their unique characteristics as organic battery components that outperform the conventional inorganic ones. Furthermore, these redox properties could be precisely tuned by using different substituent groups. Machine learning and statistics, on the other hand, have proven to be very powerful approaches for the efficient in silico design of novel materials. Herein, we demonstrated the machine learning approach for the prediction of the redox activity of quinones that potentially can serve as organic battery components. For the needs of the present study, a database of small quinone-derived molecules was created. A large number of quantum chemical and chemometric descriptors were generated for each molecule and, subsequently, different statistical approaches were applied to select the descriptors that most prominently characterized the relationship between the structure and the redox potential. Various machine learning methods for the screening of prospective organic battery electrode materials were deployed to select the most trustworthy strategy for the machine learning-aided design of organic redox materials. It was found that Ridge regression models perform better than Regression decision trees and Decision tree-based ensemble algorithms.

## 1. Introduction

In recent years, the global demand for effective energy-storage materials has constantly grown [1]. Traditionally, the widely used electrode materials in metal-ion batteries are inorganic compounds capable of reversible redox transformations [2,3]. Organic electrode materials, on the other hand, have some gainful properties, such as structural diversity and flexibility, synthetic tunability, lower price, and harmless recyclability [4,5,6,7]. Among the organic compounds considered for research on battery electrode materials, quinones have engendered the most ubiquitous expectations and extensive investigation. Quinones are a class of organic compounds derived from aromatic diols, whose redox capacity makes them interesting for designing novel organic electrode materials [8]. Quinones with a low molecular weight, such as 1,4-benzoquinone, have a relatively high redox potential [9] and, in case the two-electron redox reaction of benzoquinone takes place, a high capacity could be expected. However, due to the sublimation and dissolution of benzoquinone in the organic electrolyte solvents, a poor capacity is observed in practice [10]. These problems can be overcome by immobilizing benzoquinone on nanoparticles [11], by using various polymers containing benzoquinone fragments [12,13,14], or by introducing different substituent groups [15]. The redox potential of the quinones is dependent on the substituent type; electron-withdrawing substituents, such as carbonyl, nitro, and carboxylate groups, make quinones stronger oxidants, while electron-donating groups, such as amine, hydroxyl, and alkoxy groups, turn quinones into weaker oxidants [16]. In the present study, a dataset of quinones with electron-withdrawing substituents was constructed, since this class of materials exhibits a fairly high redox potential.

Machine learning and statistics approaches have successfully been applied for capturing the complex relationships between material structures and different properties of interest [17]. This kind of approach has also effectively been employed in the design of novel energy-storage materials: Joshi et al. [18] demonstrated that deep neural networks (DNNs), support vector regression (SVR), and kernel ridge regression (KRR) can be used to predict the redox potential of inorganic electrode materials extracted from the Materials Project Database; Zhang et al. [19] used a Crystal Graph Convolutional Neural Network (CGCNN) to creatively build an interpretable deep learning model that predicts redox potential based on inorganic crystal structures [19]. Machine learning algorithms have also been productively applied in the design of organic electrode materials: Allam et al. [20] developed a pre-screening procedure that relies on the density functional theory to compute both the redox potential of organic electrode materials and molecular descriptors, such as the electron affinity and the gap between the highest occupied molecular orbital (HOMO) and the lowest unoccupied molecular orbital (LUMO), to be used as input features of artificial neural networks (ANNs), gradient boosting regression (GBR) and KRR. A major disadvantage is that the density functional theory, which is comparatively computationally expensive, is used for descriptor computation. Tutte et al. [21] propose a Hammet-like approach to model quinone solubility in organic electrolytes that are typically used in lithium-ion batteries (the organic electrode materials must have low solubility in the battery electrolyte). Machine learning screening has also been applied for the design of quinone electrolytes for redox flow batteries [22]: Wang et al. created a dataset by generating various disubstituted quinones, replacing hydrogens in different quinone backbones with a predefined set of substituents, and, subsequently, utilized the extreme gradient boosting algorithm to build a model for screening the HOMO–LUMO gap and the free energy of solvation. In the current study, different linear and nonlinear regression models were built to predict the electrode potential of substituted quinones.

Dataset construction plays a central role in any data-driven study. In this report, two tactics for the creation of application-specific datasets were combined. Firstly, a top-down approach was used: molecular structures that satisfy some application-specific conditions (i.e., contain a quinone fragment) were extracted from PubChem [23] (a large, publicly available database). Next, a bottom-up approach was applied: the dataset of molecular structures produced in the first step was expanded via inclusion of the systematically generated derivatives of the already-selected species. This strategy guarantees that the final dataset created is structurally consistent.

## 2. Materials and Methods

### 2.1. Dataset Construction

#### 2.1.1. Molecular Structure Generation

To construct the dataset, 100 benzoquinone derivatives were extracted from the PubChem database [23] as simplified molecular-input line-entry system (SMILES) strings. The SMILES strings were converted into 3D structures using the OpenBabel software package (version 3.1.1) [24] and, subsequently, the DerGen software (version 0.1) [25] was used to generate all possible derivatives of those compounds containing a -CN or a -C≡CMe group. In total, 494 structures were produced. This dataset construction procedure guarantees that the generated molecules are structurally similar, and hence makes it easier to establish the structure–electrode potential relationship for a quinone series with electron-withdrawing substituents—a group of compounds that is particularly interesting for the design of organic energy-storage materials.

#### 2.1.2. Dataset Splitting

The dataset was shuffled and split into a training set (395 compounds, 80% of the whole dataset) and a test set (99 compounds, 20% of the whole dataset). To avoid data leakage [26], the descriptor selection and hyperparameter optimization were performed on the training set. An average R^2^ metric over 5-fold cross-validation was used for model performance assessment during the descriptor selection and hyperparameter optimization.

### 2.2. Molecular Descriptors

Representing molecular structures in an unambiguous machine-readable format is not a trivial task. Many different molecular representations have been developed [27]. Molecular structures can be represented as the following:

- Strings—for example, the SMILSES representation that contains information about atom types and connectivity [28];
- Connection table formats [29]: tabular formats that provide information about atom counts, atom types, connectivity matrix, bonded pairs of atoms, chirality, etc.; an example for such molecular representation format is the MDL molfile;
- Vectors of features: a molecule can be represented either as a vector of molecular properties (descriptors) such as molecular weight, molecular volume, numbers of certain atom types, topology, etc., [30] or as a molecular fingerprint: a bitstring (can be regarded as vector of ones and zeros) is derived from the molecular structure according to a predefined set of rules [31]—among the most employed fingerprints are the extended-connectivity fingerprints (ECFPs) based on Morgan’s algorithm [32], since they are specially designed for establishing structure–property relationships [33];
- Computer-learned representations: in recent years, a large number of machine learning-based molecular representations were developed—those methods rely on convolutional neural networks (CNNs) and/or recurrent neural networks (RNNs) to transform a molecule represented as a SMILES string or as 3D Cartesian atom coordinates to a low dimensional latent space [34,35] that can be used both for property prediction and for the generation of new molecular structures [36].

In the current study, the PaDEL [37] software package was employed to generate a multitude (750 descriptors per molecule) of cheminformatics-based molecular descriptors, and the MOPAC program suite [38] was used to produce semi-empirical descriptors such as HOMO and LUMO energies and the dipole moments of the reducible compounds.

#### Descriptor Selection

**Figure 1:**
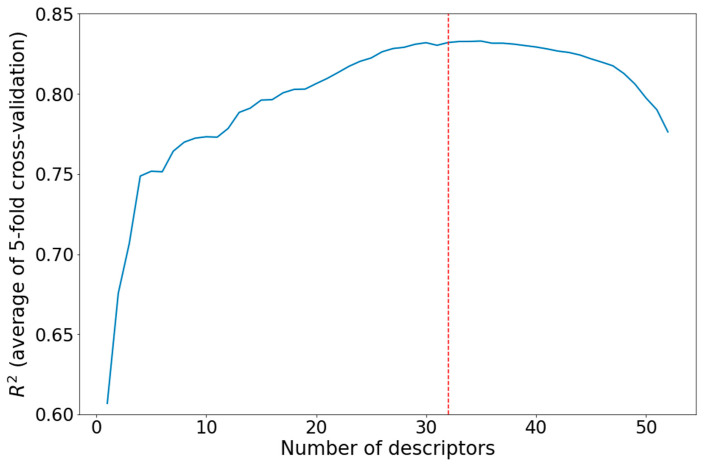
Results of the backward stepwise regression for descriptor selection.

Feature selection is a key step in any data-driven study [39]. The objective of the feature selection procedure is to assort features that have a strong correlation with the target variable. In the current work, the following steps were taken:

- Low-variance descriptors were removed: descriptors whose value equalled the descriptor mode for 60% or more of the molecules in the dataset were discarded;
- Descriptors that had a weak correlation with the target value were discarded. Correlations with covariance between the normalized descriptors and normalized target values of less than 0.25 were considered as weak correlations. The normalization was performed as follows:
  $$V_{norm}=\frac{V-V_{mean}}{\sigma_{V}},$$
  where *V_norm_* is the normalized value, *V* is the unnormalized value, *V_mean_* is the mean of *V* in the dataset, and *σ_V_* is the standard deviation of *V* in the dataset.
- Strongly mutually correlated descriptors (covariance between normalized descriptors greater than 0.7) were discarded. After this operation, 52 descriptors were left;
- Backward stepwise regression [40] was used for further descriptor reduction (Figure 1). Finally, 32 descriptors remained (Table 1).

### 2.3. Redox Potential Calculation

The redox potential was calculated with the density functional theory (DFT) for the redox reaction:


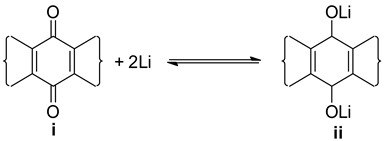


Geometry optimization was performed on all quinone derivatives in the dataset (i) and their respective reduced forms (ii) using the B3LYP functional in combination with the 6-311++G(2df,2p) basis set, as implemented in the Gaussian 16 software package (version EM64L-G16RevB.01, 20 December 2017) [48]. This protocol was chosen as a trade-off between precision and computational time.

The electrode potential was calculated using the Nernst equation:

(1) $$∆E=\frac{-∆G}{nF},$$

where *n* is the number of exchanged electrons, *F* is the Faraday’s constant, and the reaction free energy, Δ*G*, is calculated as follows:

(2) $$∆G=G_{ii}-G_{i}-2G_{Li}.$$

*G_ii_* and *G_i_* were obtained from the B3LYP/6-311++G(2df,2p) calculation, as follows [49]:

(3a) $$H_{X}=E_{0}+ZPE+H_{trans}+H_{rot}+H_{vib}+RT$$

(3b) $$S_{X}=S_{trans}+S_{rot}+S_{vib}+S_{el}$$

(3c) $$G_{X}=H_{X}-TS_{X},$$

where *E*_0_ is the total electronic energy, ZPE is the unscaled zero-point energy, *H_trans_*, *H_rot_*, and *H_vib_* are, correspondingly, the translational, rotational, and vibrational shares in the enthalpy, *S_trans_*, *S_rot_*, *S_vib_*, and *S_el_* are, respectively, the rotational, translational, vibrational, and electronic motion contributions to the entropy. RT represents the work term converting the internal energy into enthalpy (*T* = 298 K). *G_Li_* is the free energy of lithium in the gas phase. A comparison of calculated and experimental values of electrode potentials showed that when the free energy change in the redox reaction is estimated as the difference of the free energies of the reacting molecules in the gas phase, then the gas phase free energy for lithium should be considered as well (see Supplementary Information in [50]).

### 2.4. Machine Learning Methods Used

Different machine learning methods were deployed to investigate the relationship between the molecular structure and the electrode potential.

#### 2.4.1. Ridge Regression

Ridge regression is a method for estimating the coefficients of l2-regularized multiple linear regression models:

(4) Xβ=y,

where, for a dataset consisting of *n* molecules, each is represented as an *m*-dimensional vector; ***X*** is an *n_x_*(*m* + 1) matrix of *n*-dimensional column vectors ***x_j_*** (***x*_1_** is [1 1 … 1]^T^, while *x*_2_, *x*_3_, …*x*_(*m* + 1)_ are the values of the corresponding descriptors), known as explanatory variables; ***β*** is an (*m* + 1) dimensional vector of parameters, where *β*_1_ is the intercept term and ***y*** is the vector of the observed values (redox potentials in the current study). The ridge estimator of ***β*** is given using the following equation [50]:

(5) $$\beta ={\left(X^{T}X+\lambda I\right)}^{-1}X^{T}y,$$

where *λ* is a regularization coefficient and ***I*** is the identity matrix. Ridge regression is known to perform better than linear regression in cases of mutually correlated explanatory variables (molecular descriptors in our case) [51].

#### 2.4.2. Decision Tree

First introduced in 1987 [52], decision trees are hierarchical supervised machine learning models that logically combine a sequence of decisions, based on simple tests, and their possible outcomes. This is achieved by optimizing the simple test condition threshold during the training process [53]. In the course of training, all possible data splits are considered:

(6a) $$Q_{m}^{l}=\left\{\left(x,y\right)\right|x_{j}<t_{m}\}$$

(6b) $$Q_{m}^{r}=Q_{m}\;\backslash \;Q_{m}^{l},$$

where *Q_m_* is the data at node *m*, $Q_{m}^{l}$ and $Q_{m}^{r}$ are the candidate splits, ***x*** is the training data vectors, and ***y*** is the target variable vector. The threshold condition is optimized by comparing the quality of the splits using an appropriate cost function. For regression decision trees, the mean square error (MSE—Equation (8a)) or the Poisson deviance (Equation (8b)) can be used as cost functions [52]:

(7) $${\bar{y}}_{m}=\frac{1}{n_{m}}\sum_{y\in Q_{m}}y$$

(8a) $$H\left(Q_{m}\right)=\frac{1}{n_{m}}\sum_{y\in Q_{m}}{\left(y-{\bar{y}}_{m}\right)}^{2}$$

(8b) $$H\left(Q_{m}\right)=\frac{1}{n_{m}}\sum_{y\in Q_{m}}{\left(ylog\left(\frac{y}{{\bar{y}}_{m}}\right)-y-{\bar{y}}_{m}\right)}^{2}$$

This splitting operation is performed for all the features, and the feature split that leads to the largest decrease in the cost function is kept at node *m*. This allows for the estimation of the feature importance—the more efficiently a feature split decreases the cost function, the more important the feature.

It should be noted that, due to their structure of sequential simple tests, decision trees are able to capture nonlinear dependencies between the explanatory variables and the measured property. Decision trees have been successfully utilized to solve both classification and regression problems [54,55,56]. There exist numerous algorithms for decision tree construction: ID3, C4.5, CART, MARS, and CHAID [57]. In the present study, the CART (classification and regression tree) algorithm with a mean square error cost function was used.

#### 2.4.3. Random Forest

Random forests are ensemble machine learning algorithms that can be used for classification and regression. Multiple decision trees are constructed using randomly selected explanatory variables (molecular descriptors in our case) and each tree is trained on different bootstrapped samples (sampling, allowing for multiple selection of the same items) of the training set. When a prediction is made, the average result of all trees is returned [58].

#### 2.4.4. Extra Trees

The extra trees algorithm [59] is similar to the random forest algorithm—a multitude of decision trees are used; however, the individual decision trees are trained on subsamples of the training set taken without replacement (in contrast to bootstrapping). Another important difference is that, in the extra trees algorithm, the cut point is selected randomly, while in the random forest algorithm, the optimal split is chosen. These differences generally lead to the reduction of bias and variance. The random choice of a cut point also makes the algorithm faster (in the random forest algorithm, the optimal split is found by computing some impurity metric for all possible splits).

#### 2.4.5. Gradient Boosting

Gradient boosting relies on the fitting of a sequence of weak prediction models (decision trees in this case) on repeatedly altered versions of training data [60]. The predictions of all individual weak predictors are combined as a sum:

(9) $${\hat{y}}_{i}=F_{M}\left(x_{i}\right)=\sum_{m=1}^{M}h_{m}(x_{i}),$$

where *ŷ_i_* is the model prediction, ***x****_i_* is a vector of all features that describes the *i*-th object in the dataset (in our case, all descriptors used to represent a molecule), *M* is the number of weak estimators, and *h_m_*(***x****_i_*) is the prediction of the *m*-th weak estimator. From Equation (9), it follows that

(10) $$\;F_{m}\left(x_{i}\right)=\;F_{m-1}\left(x_{i}\right)+h_{m}(x_{i}).$$

The weak predictor *h_m_*(***x****_i_*) in Equation (10) is fitted to minimize a sum of the cost functions, *Cm*:

(11) $$h_{m}={argmin}_{h}\left(C_{m}\right)={argmin}_{h}(\sum_{i=1}^{n}c(y_{i},\;{\;F}_{m-1}\left(x_{i}\right)+h_{m}(x_{i}))),$$

where *n* is the number of training entries and *c*(*y_i_, F*(***x****_i_*)) is a cost function, such as the mean square error (MSE, Equation (8a)).

Friedman [59] proposed a regularization strategy, based on scaling the contribution of each new weak predictor, based on a learning rate (*γ*):

(12) $$F_{m}\left(x_{i}\right)=\;F_{m-1}\left(x_{i}\right)+\gamma h_{m}(x_{i}).$$

It has been demonstrated [61] that, in many cases, gradient boosting outperforms other ensemble methods such as random forests and extra trees.

In the present study, all machine learning algorithms were exploited as implemented in the scikit-learn library [62].

## 3. Results and Discussion

**Figure 2:**
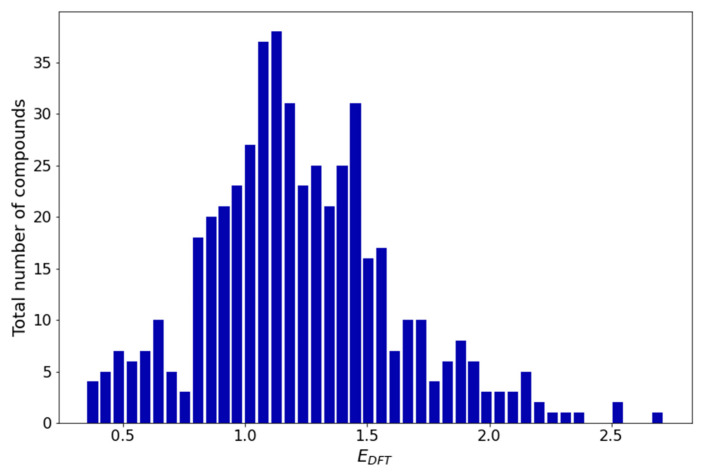
Redox potential distribution histogram.

The redox potential distribution (Figure 2) over the entire dataset (494 compounds) shows that the redox potential spans the range of 0.3–2.8 V. The distribution plot has an asymmetric bell-like shape, with the majority of compounds having potentials between 0.75 V and 1.60 V.

In order to find an optimal approach for the machine learning modelling of structure–redox potential relationships, the following algorithms were tested: ridge regression, decision tree, random forest, extra trees, and gradient boosting. Artificial neural networks were not considered, since they are prone to overfitting, especially when trained on an insufficient amount of data [63].

In order to attain maximal predictive ability, the hyperparameters (parameters that control the learning process) of each of the machine learning models were optimized using a grid search. The model performance was evaluated based on the averaged coefficient of determination (R^2^) [64] of the five-fold cross-validation over the training set. The training R^2^ was also taken into account, since the difference between the validation and training R^2^ can be used to judge whether the model is overfitted.

**Figure 3:**
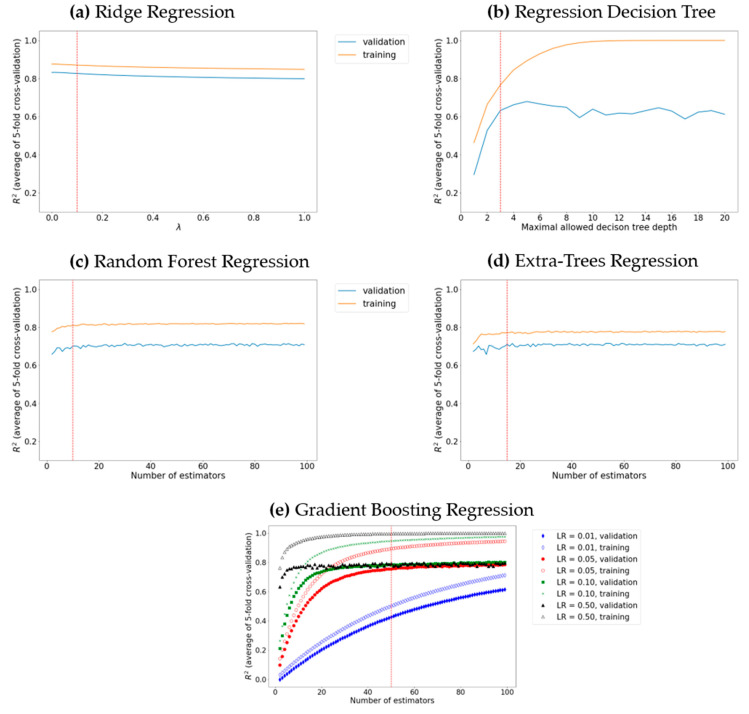
Model tuning via grid search for (**a**) the optimal learning rate in ridge regression; (**b**) the optimal maximal decision tree depth; ((**c**) and (**d**), respectively) the optimal number of decision tree estimators in random forest regression and extra trees regression; (**e**) the optimal number of decision tree estimators and the learning rate (LR) in gradient boosting. The optimal hyperparameter value is depicted by a dotted red line.

The l2-regularization value (*λ* in Equation (5)) in ridge regression does not have a significant impact on the model performance (Figure 3a); increasing the l2-regularization value leads to a decrease (by an almost equal amount) of the training and validation R^2^. It should be noted that the difference between the training and validation R^2^ reached a minimum at *λ* = 0.1, and hence, this value of lambda results in an optimal (neither underfitted, nor overfitted) ridge regression model.

**Figure 4:**
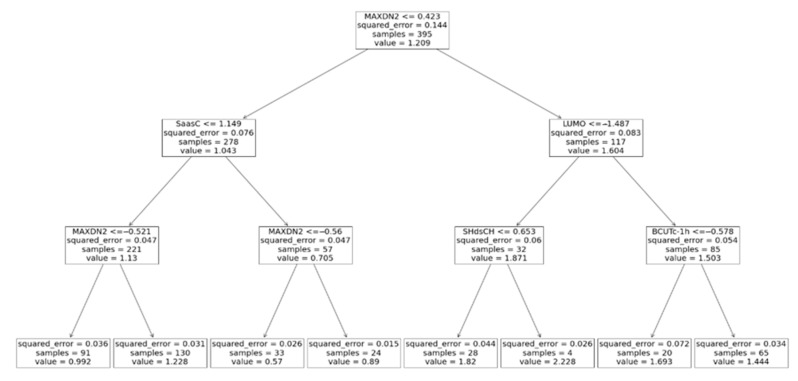
Regression decision tree chart with maximal depth of three.

The decision tree maximal allowed depth plays a central role in determining whether the decision tree underfits or overfits the training data: a larger maximal allowed depth results in a deeper tree that fits the training data better; however, if a tree is too deep, the noise in the training data is also learned, i.e., the decision tree overfits. In the present work, the maximal tree depth varied from two to twenty (Figure 3b). Optimal algorithm performance was attained when the maximal tree depth was three. A serious advantage of decision trees is their ability to visualize the learning process (Figure 4). Furthermore, the decision tree algorithm enables the examination of the descriptor significance. It was found that the most significant descriptors, MAXDN2, LUMO, SaasC, SHdsCH, and BCUTc-1H (see Table 1), are all related to the electronic structure of the molecules—quinones that contain more CN and C≡C-Me groups (lower LUMO, large MAXDN2 due to CN groups) exhibit larger redox potential.

To examine the predictive ability of maximal random forest regression and extra trees regression, the depth of the decision tree estimator was set to three (since we established that this value of maximal depth ensures maximal learning performance), and the number of estimators was optimized to achieve the maximal coefficient of determination over the validation set (Figure 3c,d): 10 and 15 estimators were chosen for random forest and extra trees, respectively.

It was found that the extra trees algorithm is less prone to overfitting: the R^2^ value over the validation set is closer to the R^2^ value over the test set. The random forest and extra trees algorithms can also be used to estimate the descriptors’ importance—the most significant descriptors for the decision tree (described above) are found among the ten most significant descriptors of both algorithms, which confirms that the descriptors related to the electronic structure, such as the LUMO energy, and descriptors derived from electronegativity, such as SaasC, SHdsCH, MAXDN2, and meanI, are important for the machine learning prediction of the redox potential of organic energy-storage materials. As expected, we found that the gradient boosting regression exhibits a better predictive ability and is less prone to overfitting than the other ensemble methods used (random forest regression and extra trees regression). The learning rate (γ in Equation (12)) and the number of weak predictors values of 0.05 and 50, respectively, were found via grid searching (Figure 3e).

The prediction models’ performance, as evaluated based on the average coefficient of determination over a five-fold cross-validation (R^2^_CV_), increases in the following order: regression decision tree (R^2^_CV_ = 0.632), random forest regression (R^2^_CV_ = 0.705), extra trees regression (R^2^_CV_ = 0.715), gradient boosting regression (R^2^_CV_ = 0.756), and ridge regression (R^2^_CV_ = 0.832).

**Figure 5:**
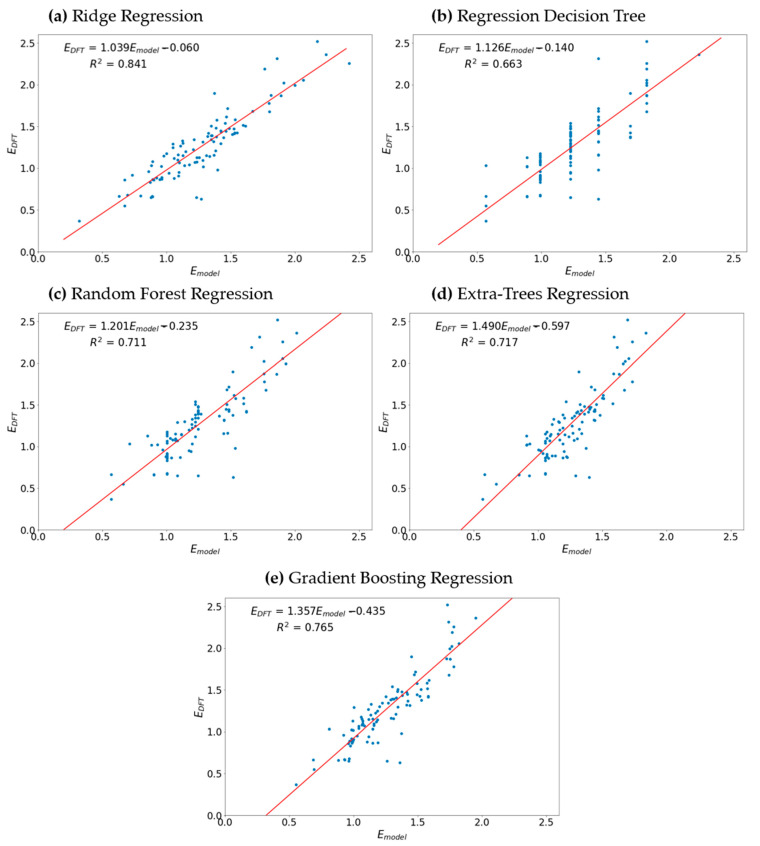
Models’ performance on the test set.

All machine learning algorithms were evaluated on the test set. To visualize the model performance, scatter plots of the redox potential calculated based on the density functional theory versus the redox potential estimated using the corresponding machine learning algorithms were drawn (Figure 5). Linear regression was implemented to construct a trendline in the (E_model_, E_DFT_)—space (Figure 5, red lines), and the slope, intercept, and coefficient of determination (R^2^) of the trendline were calculated. When the model ideally fits the data, the trendline slope is supposed to have one and zero for the slope and trendline, respectively, and the R^2^ value should be close to one. It was found that the models’ performance on the test set does not differ significantly from the models’ performance observed upon the five-fold cross-validation, which means that the models fit the data fairly well (i.e., the models are not significantly overfitted or underfitted). All models tend to give worse prediction for large voltages: a possible explanation is that, in the training set, there are fewer molecules exhibiting a high redox potential. The dataset and machine models’ source code are publicly available: https://github.com/carim2020/org-redox-dataset (accessed on 12 October 2023).

## 4. Conclusions

We have constructed a dataset of 494 potential organic electrode materials through the automated generation of derivatives of 100 quinones, extracted from a general-purpose public database (PubChem). A descriptor selection procedure that combines low-variance descriptor removal with covariance matrix analysis and stepwise linear regression for finding uncorrelated descriptors, on which the redox potential of the molecules in the dataset depends, was devised. Due to the comparatively small dataset size, deep learning approaches were not deployed as inappropriate, since they are prone to overfitting when trained on small amounts of data. Five different supervised machine learning models for regression that tend to give better results for smaller datasets were built. The hyperparameters of all those models were tuned to attain the maximal electrode potential predictive ability. The models’ performance was evaluated on a test set containing molecules that are completely unknown to the model. It was established that the model performance increases in the following order: regression decision tree < random forest regression < extra trees regression, gradient boosting regression < ridge regression. It turned out that the linear model, i.e., the ridge regression, outperforms the decision tree-based algorithms, known to be able to capture nonlinear dependencies between the descriptors and the target variable. This is an implication that the relationship between the electrode potential and some chemical properties is most probably linear. In particular, it was found that descriptors related to the electronic structure (LUMO and E-state descriptors) have a large significance. In addition, ridge regression is an excellent method for the screening of databases, as it is a very fast and computationally inexpensive approach, compared to other machine learning algorithms.

## Figures and Tables

**Table 1:** Molecular descriptors’ names and descriptions.

Description	Descriptor Name
Lowest partial charge weighted BCUTS [41]	BCUTc-1l
Highest partial charge weighted BCUTS [41]	BCUTc-1h
Total number of double bonds (excluding aromatic bonds)	nBondsD2
Triply bound carbon bound to another carbon	C1SP1
Doubly bound carbon bound to three other carbons	C3SP2
A topological descriptor combining distance and adjacency information [42]	ECCEN
Count of atom-type H E-State: H on aaCH, dCH2 or dsCH * [43]	nHother
Count of atom-type E-State: =C< [43]	ndssC
Count of atom-type E-State: aaC- [43]	naasC
Count of atom-type E-State: N≡ [43]	ntN
Sum of E-States for weak hydrogen bond acceptors [43]	SwHBa
Sum of atom-type H E-State: =CH- [43]	SHdsCH
Sum of atom-type H E-State: H on aaCH, dCH2 or dsCH [43]	SHother
Sum of atom-type E-State: =C< [43]	SdssC
Sum of atom-type E-State: aaC- [43]	SaasC
Sum of atom-type E-State: N≡ [43]	StN
Minimum atom-type H E-State: H on aaCH, dCH2 or dsCH [43]	minHother
Minimum atom-type E-State: aaC- [43]	minaasC
Minimum atom-type E-State: =O [43]	mindO
Maximum atom-type H E-State: H on aaCH, dCH2 or dsCH [43]	maxHother
Maximum atom-type E-State: aaC- [43]	maxaasC
Mean intrinsic state values I [43]	meanI
Maximum negative intrinsic state difference in the molecule (related to the nucleophilicity of the molecule) [44]	MAXDN2
Maximum positive intrinsic state difference in the molecule (related to the electrophilicity of the molecule) [44]	MAXDP2
Complexity of the system [45]	fragC
Number of rings	nRing
Topological diameter (maximum atom eccentricity)	topoDiameter
Mean topological charge index of order two [46]	JGI2
Topological polar surface area	TopoPSA
Van der Waals volume calculated using the method proposed in Zhao et al. JACS 2003, 68, 7368–7373 [47]	VABC
Molecular weight	MW
Energy of the lowest unoccupied molecular orbital estimated by PM6 [eV]	LUMO

* a = aromatic; s = single; d = double.

## Data Availability

The computed data are available from the authors on request.
